# (Ascorb)ing Pb Neurotoxicity in the Developing Brain

**DOI:** 10.3390/antiox9121311

**Published:** 2020-12-21

**Authors:** Faraz Ahmad, Ping Liu

**Affiliations:** Department of Anatomy, University of Otago, Dunedin 9016, New Zealand; ping.liu@otago.ac.nz

**Keywords:** lead, ascorbate, neuronal, antioxidant, chelation, nutrient, vitamin C, dehydroascorbate, blood lead level (BLL)

## Abstract

Lead (Pb) neurotoxicity is a major concern, particularly in children. Developmental exposure to Pb can alter neurodevelopmental trajectory and has permanent neuropathological consequences, including an increased vulnerability to further stressors. Ascorbic acid is among most researched antioxidant nutrients and has a special role in maintaining redox homeostasis in physiological and physio-pathological brain states. Furthermore, because of its capacity to chelate metal ions, ascorbic acid may particularly serve as a potent therapeutic agent in Pb poisoning. The present review first discusses the major consequences of Pb exposure in children and then proceeds to present evidence from human and animal studies for ascorbic acid as an efficient ameliorative supplemental nutrient in Pb poisoning, with a particular focus on developmental Pb neurotoxicity. In doing so, it is hoped that there is a revitalization for further research on understanding the brain functions of this essential, safe, and readily available vitamin in physiological states, as well to justify and establish it as an effective neuroprotective and modulatory factor in the pathologies of the nervous system, including developmental neuropathologies.

## 1. Introduction

Lead (Pb) is a metallic contaminant. Though it is not a particularly abundant element, Pb contamination is prevalent in all phases of the environment (air, water, soil, and food chain). Hence, its exposure can be mediated through all these sources. Pb is among the oldest known occupational toxins. However, due to its widespread employment in the manufacturing of batteries, paint pigments, alloys for solder, ammunition, and plastics; the commercial usage of Pb has exceeded all other previous periods in the 20th century. This has resulted in a significant mobilization of free Pb in the environmental and biological spheres, with a consequent increase in its potency as a toxicant. Of note, almost all forms (metallic, organic, and inorganic) of Pb can be equally potent in their toxicities. Unfortunately, Pb is also nonbiodegradable and as such has a prolonged persistence. Because it detrimentally effects a broad-range of physiological, biochemical, and behavioral functions, Pb is a well-studied toxicant [1]. In fact, it affects almost all tissues and organ systems, such as the respiratory, hematopoietic, renal, cardiovascular, urinary, and urogenital systems, as well as bones [2,3,4,5]. Moreover, apart from its widely characterized effects in humans [4,5,6,7,8] and animals [3], the detrimental effects of Pb on the physiology of plants [9] and fish [10,11,12] have also been well-documented.

## 2. Pb Neurotoxicity in Children

The brain is a prominent target of Pb toxicity. The developing brain is particularly vulnerable to exposure to environmental neurotoxicants such as Pb [13,14,15,16]. Interestingly, contemporary researchers have repeatedly documented no safe blood lead level (BLL) in children [15,17,18], indicating that any early-life exposure to Pb, even in minute quantities, leads to sustained neuropathological alterations [19,20,21,22]. Because of the disproportionate incidence of Pb exposure in children, it is not surprising that the majority of epidemiological research on the health effects of Pb has been focused on children [13,15,23,24]. There are a number of factors responsible for this. First, children exhibit an increased gastrointestinal absorption of Pb [7,25]. Second, their behavior and lifestyle (more hand-to-mouth activities and outdoor time, as well as physical proximity to ground level) increase the likelihood of Pb exposure from contaminated soil and dust [19]. Moreover, children’s immature nervous system is particularly vulnerable to Pb toxicity because of a developing blood–brain barrier (BBB) [6,26]. The neurodevelopmental phenomena of cell division, migration, synaptogenesis, synapse pruning, and the formation of neuronal–glial interactions occur during critical periods in fetal and early childhood, and Pb can suppress all these processes [7,26]. It is therefore not surprising that early-life Pb exposure can have detrimental effects on neurodevelopmental trajectory and long-lasting implications for neurocognition [7,26,27]. It should be noted that Pb neurotoxicity is exacerbated by concomitant exposure to other neurotoxicants [28]. For the purpose of this review, however, we solely concentrate on the Pb-mediated effects. Interestingly, Pb exposure in children can also reduce the intake of essential nutrients, as indicated by a strong negative correlation between BBLs and dietary iron intake [29,30], signifying the potency of the multiple and sometimes indirect effects of Pb exposure.

### 2.1. Sources of Pb Exposure in Children

Sources of Pb exposure are mainly environment-dependent but are highly varied. The exposure of children from mothers who have been exposed occupationally [31], for example, can be through the placenta in utero and through breast milk [32,33,34,35,36]. Dwelling in geographical locations, such as mines and smelters [37,38], industrial sites [39,40], and waste sites [41], have also been known to result in significant elevations in BLLs in children. Socioeconomic status is another key determinant that increases the likelihood of early-life exposure to Pb [40,42,43]. This might be due to low nutritional status and increased likelihood of exposure to Pb, e.g., from the air or in low income accommodation containing lead paints [7,44]. In particular, a poor diet and deficiency of essential nutrients, such as milk products, are critical predictors of the Pb burden and BLLs [45]. Parental and community factors, such as parental education and occupation, number of siblings, standard of living indices, living in a crowded neighborhood, exposure to tobacco smoke, and playing outdoors, might also explain the association between socioeconomic status and Pb burden in children [37,46,47,48]. Interestingly, recreational activities like shooting have also been shown to result in Pb exposure and elevated BLLs in humans, including high school students [49]. Moreover, dietary exposure to Pb in wild gamebirds shot with lead ammunition significantly increases BLLs in children concomitantly with reductions in intelligence quotient (IQ) levels [50]. Lastly, man-made disasters, such as wars and military assaults on civilian populations, contribute tremendously to the likelihood of heavy metal neurotoxicity in children. This is particularly concerning for the besieged Palestinian population in the Gaza Strip who have endured three successive wars in the recent years [51,52,53,54]. 

It is of interest to note that genetic factors can predispose children to Pb-mediated effects on neurodevelopment and behavioral outcomes. Polymorphisms in the δ-aminolaevulinic acid dehydratase (ALAD) and vitamin D receptor (VDR) genes may potentially modify the effects of Pb exposure [55]. Gender is another critical factor that might contribute to the Pb burden and its effects in events of early-life exposure [56,57]. 

Conclusively, while this means that Pb neurotoxicity in children is dependent on a number of environmental factors (and genetic factors to some extent), considerable efforts are needed to reduce the risk of exposure at several levels [14,28,36,58,59]. Legislative measures, particularly concerning leaded gasoline, have indeed aided in reducing the incidences of Pb exposure [60,61,62]. Other factors such as improved protocols for pediatric screening by primary care givers might also prove to be helpful in this regard [63].

### 2.2. Neurobehavioral Effects of Early-Life Pb Exposure

Widespread studies in diverse environments have been conducted to understand the physiological implications of Pb exposure and elevations in BLLs in children. Multiple effects have been found to underlie the pathophysiology of Pb intoxication in children that manifest early in the subjects’ lives and predisposes them to development of neurological and psychological diseases much later in adulthood [7,26].

General cognition outcomes, as well as IQs, have been found to be correlated with BLLs in children [7,19], and the severity of deficits as measured by decrements in IQ points parallels the elevations in BLLs [22,64]. The steepest decrements in IQs have been observed to occur at BLLs in excess of <100 μg/L [19]. Fetal exposure to Pb has also been observed to induce an adverse effect on neurodevelopment, with observable deficits even at two years of age [35]. Similarly, a prospective study showed that a low exposure to Pb early in the life at 18–30 months of age resulted in a persistent decrease in IQ up to four-to-six years of age [65]. Consistent with the notion that early-life Pb exposure can have detrimental effects on neurodevelopmental trajectory and long-lasting implications for neurocognition, Baghurst et al. reported prenatal Pb exposure to have a lingering inverse effect on IQ levels for children up to seven years old [66] and further effects at up to 11–13 years [67] in children of the lead smelting community of Port Pirie, Australia. In a nine-year prospective study conducted in children based in Taiwan, BLLs were found to be significantly correlated with decreasing IQs, as well as delayed cognitive development, at five-to-eight years of age [68]. An inverse association of intellect function in children measured by an IQ-based assessment with BLL was found in a population of Italian adolescents [69]. Apart from general cognition, a strong inverse relationship between postnatal Pb exposure and executive functioning and goal-oriented problem solving (involving working memory, cognitive flexibility, attention/inhibition, and unitary executive functions) in children has been observed [21,70]. Even infants aged less than six months with prenatal exposure to Pb elicited strong neurobehavioral abnormalities [28]. Other aspects of child intelligence that are adversely affected by pre- and post-natal exposure to Pb include reading/language and arithmetic skills [56,71,72,73], nonverbal reasoning [32], reaction times [72], visual–auditory integration, visual–motor integration, and fine motor skills [71,74,75], as well as short term memory [7,70,71,72]. Lastly, a prospective cohort study in a New Zealand population born in 1972–1973 with possibly the longest follow-up period (four decades) to assess the effects of early-life Pb exposure confirmed the persistent effects of childhood Pb exposure, as indicated by a strong correlation between childhood BLLs with deficits in cognitive functions (perceptual reasoning, working memory, and IQ) and decline in socioeconomic status at 38 years [76].

Exposure to environmental and dietary Pb has been proposed to be a significant risk factor for attention deficit hyperactivity disorder (ADHD) in children, which is characterized by inattention, impulsivity, and hyperactivity [77,78]. Thus, using a child behavior assessment rating scale, Boucher et al. found that low levels of childhood Pb exposure were associated with ADHD behavior [79]. Other studies have also proposed a significant link between Pb burden, clinical ADHD cases, and AHDH-like behavior in children [28,70,80,81]. Interestingly, children with medically diagnosed AHDH conditions and controls dwelling near the Pb investigation area of a former refinery showed an association of BLL (but not blood Hg and Cd levels) with the disorder, indicating an elevated risk due to postnatal Pb, but not Hg or Cd, exposure [82]. Consistent with these findings, a questionnaire- and performance-based assessment of Romanian children found significant associations of ADHD-like behavior with BLL but not with other toxic metals, notably Hg and Al [83]. Interestingly, animal studies have also provided evidence for similar hyperactivity and attention deficits in rodents exposed to Pb [84,85,86,87].

ADHD-like behavioral attributes are considered as major risk factors for delinquent behavior including drug abuse later on in life. Indeed, childhood Pb exposure has been observed to be associated with substance abuse as adolescents [88,89]. Elevated Pb burden, as assessed by bone [90] and blood [89] Pb levels, has been associated with aggression and antisocial behavior in arrested juveniles. An association of delinquency, as measured by hostility, disruptive behavior, and difficulty in emotional regulation and processing of emotional cues with low BLLs, has also been observed in 9–11-years-old children from the Environmental Exposures and Child Health Outcomes (EECHO) project in the US [91]. An association of emotional problems in children with BLLs was also proposed in an independent study [92]. Predisposition of children to neuropsychiatric stress and anxiety by Pb exposure has been confirmed in a study conducted on young children in Chennai, India; wherein higher incidence of anxiety was observed as a function of BLL [70]. Delinquent behavior and emotional problems are often found to be associated with disruptions in signaling at the hypothalamus pituitary adrenal (HPA) axis [93,94]. Not surprisingly, models of developmental Pb exposure in animals have supported the hypothesis of neurodevelopmental alterations in emotional regulation via functional alterations in the HPA axis response system [95,96,97,98].

The incidence of autism spectrum disorders (ASD) in children is also often associated with early-life Pb exposure and elevations in BLLs [28,58,99]. In fact, the degree of autism severity may strongly correlate with Pb levels in hair and nail samples [100]. Schizophrenia is another disease that is heavily influenced by gene–environment interactions. There is evidence that early-life exposure to Pb can predispose individuals to develop psychiatric conditions like schizophrenia later in the life [101,102,103]. Indeed, developmental Pb exposure in experimental animals recapitulates certain behavioral and neuropathological characteristics of schizophrenic conditions, such as the disruption of the ontogenetic switch of *N*-methyl-D-aspartate receptor (NMDAR) subunits, the selective loss of parvalbumin-positive γ-aminobutyric acid (GABA)ergic interneurons, and the hyperactivity of subcortical dopaminergic system [104,105]. Moreover, Pb exposure in mutant disrupted-in-schizophrenia 1 (mDISC1) has provided further electrophysiological and behavioral evidence in support of developmental Pb as a risk factor for neuropsychiatric conditions [106].

Alzheimer’s disease (AD) is among the most common causes of dementia in the aged population. Noteworthy, most AD cases are sporadic in nature and influenced by a range of genetic and environmental factors. Recent epidemiological studies suggest an association of Pb burden and AD dementia (reviewed in [107,108]). Childhood Pb exposure, particularly in the critical period, leads to permanent alterations in nervous system functions, and this might extend to predisposing the brain to ageing-induced neurodegeneration, such as AD [109]. Indeed, evidence from studies conducted in zebrafish, rodent, and non-human primate models by Zawia’s and other research groups has supported the proposition that developmental exposure to Pb can result in an increased risk of developing AD pathology later on in life. Importantly, developmental Pb can result in alterations (at protein and gene levels) of the major players involved in the regulation of both of the major pathogenic species of AD (amyloid-beta and hyperphosphorylated tau) [110,111,112,113,114,115,116,117,118,119,120,121,122]. 

## 3. Molecular and Cellular Mechanisms of Developmental Pb Neurotoxicity

The neuropathological pathways of Pb toxicity in the developing brain have been extensively studied. Pb^2+^ mimics and substitutes for essential divalent cations like Ca^2+^ and Zn^2+^. In fact, the cellular entry of Pb^2+^ is dependent on Ca^2+^-permeant channels [18]. Because most of its deleterious effects on cellular physiology stem from its ability to substitute essential divalent cations like Ca^2+^ and Zn^2+^, Pb can influence a plethora of wide-ranging aspects of cellular physiology [18]. In the brain, particularly the developing brain, Pb can affect almost all of the processes critical to neuronal functions, such as pre- and post-synaptic functions, redox homeostasis, calcium regulation, protein homeostasis and signaling, epigenetics, genotoxicity and gene expression regulation, neuroinflammation, lipid metabolism, mitochondrial functions, and metabolism. Because there are several excellent reviews extensively describing the molecular and cellular aspects of Pb neurotoxicity, both alone [8,123,124,125,126,127,128,129,130,131,132,133,134] and as a component of toxic metal mixtures [135,136,137], we do not attempt to summarize them again in this review.

## 4. Ascorbic Acid (AA): Structure–Function

Ascorbic acid (AA) or vitamin C is water-soluble hexanoic sugar acid required for the normal healthy functioning and repair of every tissue type, mainly due to its function as a key endogenous antioxidant and a cofactor for enzymatic reactions [138,139]. Many animals can produce AA from glucose in the liver. However, because of the lack of a functional enzyme in higher primates including humans, AA is not synthesized endogenously and, hence, is an essential vitamin [140]. Significant presence in many fruits and vegetables commonly consumed by humans means that the physiological requirement for AA can normally be met satisfactorily. However, things can get complicated in cases of pathophysiological insults, a befitting example being that of scurvy [141].

**Figure 1:**
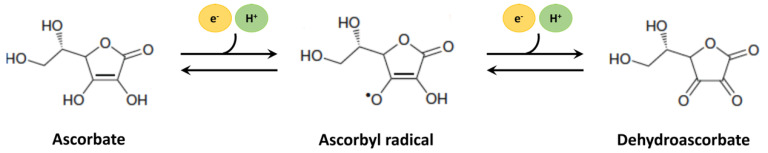
Ascorbate oxidation and recycling. Ascorbate is converted to an ascorbyl radical or semi-dehydroascorbate upon the loss of an e^−^, which is further oxidized to dehydroascorbate. Both the ascorbyl radical and dehydroascorbate can be recycled back to a monovalent, negatively-charged ascorbate ion by the action of enzyme coupled reactions utilizing thiol-based antioxidants such as glutathione and thioredoxin.

At physiological pH, AA occurs as a monovalent anion, ascorbate. As a lactone with an enediol group, it can donate electrons to convert to its neutral form, dehydroascorbate, which is formed upon the two-electron oxidation of ascorbate (Figure 1). Most biologically relevant oxidizing free radicals, however, result in one electron oxidation of ascorbate to form an ascorbyl radical or semi-dehydroascorbate. The ability to undergo transition to semi-dehydroascorbate by the action of free radicals actually forms the basis for the antioxidant property of AA and its capability to scavenge free radicals. Moreover, because ascorbate has a low redox potential, it can act as a broad-spectrum scavenger against free radicals such as peroxyl- and hydroxyl-radicals, superoxide, singlet oxygen, and peroxynitrite [142,143,144,145]. The recycling of both dehydroascorbate and semi-dehydroascorbate into ascorbate occurs by the action of glutathione and other intracellular thiol redox systems such as thioredoxin [146,147,148,149]. The antioxidant property of AA is singularly important for brain tissues because of a high rate of energy consumption, a high rate of metabolic activity, and a high polyunsaturated fatty acid content, all of which make it particularly vulnerable to oxidative damage. It is noteworthy that the antioxidant free scavenging activity of AA is not limited to the aqueous phase but also includes the protection of membranes and hydrophobic compartments through interaction with vitamin E [150,151], making it the first line of antioxidant defense [140].

The electron donating ability of ascorbate is also responsible for its ability to serve as an enzyme cofactor for redox-coupled reactions in collagen biosynthesis [152,153], noradrenaline–adrenaline synthesis [154] and biosynthesis of neuroendocrine peptides [155]. It should be noted that AA can act as a pro-oxidant in vitro and in the active sites of biosynthetic enzymes by virtue of acting as a reductant for redox-active transition metal ions such as ferric and cupric ions [156]. However, free ferric and cupric ions that are required for the pro-oxidant effects of AA are largely sequestered in forms unable to catalyze free radical reactions, at least in the healthy body [156,157]. Indeed, the in vivo pro-oxidant activities of AA, even in the presence of free, catalytically-active metal cations, have not been confirmed in physiological conditions [140,156,157]. In this respect, it should be noted the debate for in vivo pro-oxidant effects of AA have mainly relied upon a controversial study entitled “Vitamin C exhibits pro-oxidant properties” published in Nature, which reported elevated levels of DNA damage markers in human volunteers supplemented with AA [158]. However, serious questions have been raised regarding the results and their implications [159,160]. Furthermore, two major observations have supported the predominant role of AA as an antioxidant in vivo. First, AA is depleted in pathological states associated with an oxidative stress, and second, the lethality of glutathione deficiency in newborn rodents can be prevented by high doses of AA. Not surprisingly, animal and human supplementation studies have consistently supported an antioxidant function of AA with and without oxidative challenge, as evidenced by an evaluation of the markers of oxidative damage to DNA, lipids, and proteins [157]. Lastly, it should be noted here that in vitro pro-oxidant effects are not limited to AA. Some well-known reducing agents, such as glutathione and plant flavonoids, have often been found to exert damaging pro-oxidant effects when mixed with ferric or cupric ions [156,161,162].

## 5. Safety of AA

Upon the dietary intake of AA, its plasma concentration is tightly controlled at <100 μM in humans [163]. Increased amounts of ingested AA do not lead to a proportional increase in plasma AA levels; instead, it reaches a maximal upper limit due to a reduction in its intestinal absorption and an increase in its renal clearance [164,165]. While this may mean that there is no pharmacokinetic justification of using high doses of ascorbic acid, it also indicates that AA administration, even in megadoses, is practically harmless [164,166,167,168,169,170,171]. In fact, the safety of AA can be judged from the fact that the consumption of 10,000 mg AA daily for three years does not lead to any adverse health effects [172]. Moreover, claims of adverse effects of AA supplementation in higher doses have not been substantiated. For a more detailed comprehension of the pharmacokinetics of AA, readers are suggested to refer to an excellent recent review [173].

It should, however, be noted that recent studies have suggested that sustaining higher plasma levels of AA may be possible by using the oral infusion of liposomal AA [174]. Interestingly, recent studies have outlined the preparation of novel oral liposomal formulations of AA with increased bioavailability and improved antioxidant efficiency [175,176].

## 6. AA in the Brain

While AA is distributed throughout the body, the brain has the highest levels of and greatest retention capacity for AA [177,178,179,180], particularly the fetal brain [181]. Compared to the AA levels of 50 μM in plasma, AA is present at concentrations of 500 μM in cerebrospinal fluid (CSF) and 200–400 μM in extracellular fluid (ECF), highlighting presence of an active AA uptake in the brain. Indeed, the major carrier mechanisms for AA are the secondarily active specific transporters, the sodium-dependent ascorbate transporters (SVCT1 and SVCT2). While SVCT1 is responsible for the absorption of AA in the gut and its renal retention [138,140], SVCT2 has a high expression in the central nervous system (CNS), including the epithelial cells of the choroid plexus, and manages the uptake of AA from the blood into the CSF [182,183,184]. AA may also accumulate in extracellular fluid via simple diffusion across the BBB [185]. Moreover, ascorbate entry across the BBB is thought to occur by facilitative glucose transporter 1 (GLUT1), which requires the conversion into its neutral oxidation product, dehydroascorbate [186]. Dehydroascorbate is reconverted to ascorbate once inside the cells [187]. This route, however, may only be a minor pathway for ascorbate transport [183].

**Figure 2:**
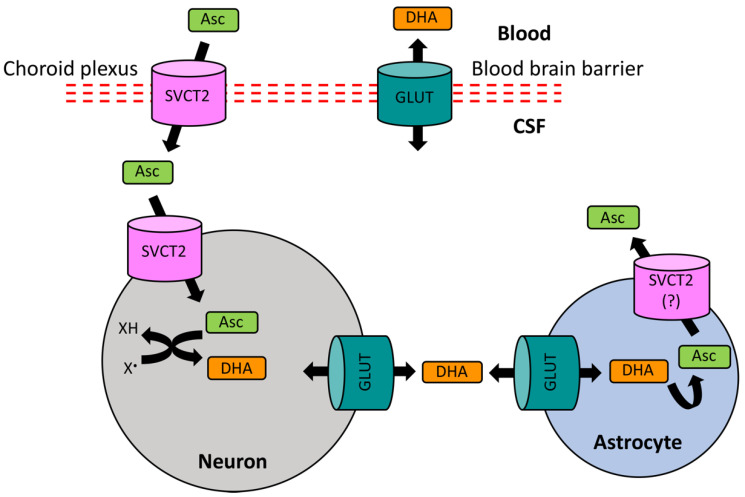
Ascorbate uptake and metabolism. Asc: Ascorbic acid in its anionic ascorbate form; GLUT: glucose transporter; SVCT: sodium-dependent ascorbate transporter; CSF: cerebrospinal fluid; DHA: dehydroascorbate; X^•^: free radical species; XH: reduced/scavenged radicals. Ascorbic acid enters the CSF in its anionic ascorbate form (Asc) through the choroid plexus via SVCT2 in the epithelial cells and as dehydroascorbate (DHA) through the GLUTs across the BBB. The entry of Asc in neurons is also mediated by SVCT2, wherein it scavenges various free radicals (X^•^) upon its oxidation to DHA. Neuronal DHA is released into the extracellular fluid for uptake by astrocytes via the GLUT-mediated transport. DHA is then recycled back to Asc for its own need or release into the extracellular space for uptake by neurons.

From the CSF, AA equilibrates with ECF and is accumulated in neurons and glia, where its concentration can reach up to 1 mM in glia and 10 mM in neurons [178,188]. The uptake of AA by brain cells is again mainly mediated by SVCT2 [184,189]. Neuronal and ECF levels of ascorbate can also be regulated by a glutamate–ascorbate hetero-exchange mechanism, possibly involving glutamate receptors [190,191]. In general, ascorbate content in neuron-rich grey matter is much higher than that in white matter, which is evident from its higher levels in anterior brain regions (cerebral cortex and hippocampus) and progressively lower levels in posterior regions (brain stem and spinal cord) [178]. There is also evidence that astrocytes rely on the GLUT-mediated transport of dehydroascorbate for regulating their intracellular AA levels [189,192]. Since, unlike the active SVCT-mediated ascorbate transport, the facilitated diffusion of dehydroascorbate via GLUTs is bidirectional, glial cells can regulate the levels of dehydroascorbate both intracellularly and in the vicinity of the synaptic contacts [138,193]. This is particularly important in the activity-induced brain state. Upon the stimulation of neuronal activity, AA is released from glial reservoirs into extracellular space [194,195]. AA in the synaptic cleft is taken up by neuronal terminals using SVCT2 (Figure 2) [196], where it is utilized for scavenging oxidizing free radicals. The consequently formed dehydroascorbate is then released in to the extracellular space by GLUTs [193,197], from where it is taken up by astrocytes through facilitated diffusion via GLUTs [193,197] and is then recycled back to ascorbate using glutathione and other antioxidant systems [140,198]. To this end, it seems logical to assume that a high concentration of AA in the brain (particularly in neurons) is an allostatic measure to match the higher oxidative metabolism rate, thus indicating that AA may act as a major endogenous antioxidant that maintains redox homeostasis [140,199]. Indeed, neurons have a much higher oxidative metabolic rate and lower level of redox resistance relative to glia [200]. In fact, neurons may be particularly sensitive to AA deficiency because of higher rates of metabolic activity [138,196].

From the neuronal perspective, AA can act both as a neuromodulator [201,202,203] and a neuroprotectant [139,178,180]. The modulation of neurotransmission by AA is known to occur at cholinergic, catecholaminergic, and glutamatergic synapses, wherein it can influence neurotransmitter release, binding, and uptake, and serves as a cofactor for their biosynthesis [204]. Neuroprotection mediated by AA in neuropathological states also occurs in a multimodal fashion. Apart from its described-above antioxidant functions, AA has anti-inflammatory and anti-depressive functions, as well as modulatory actions on epigenetic and gene expression mechanisms (reviewed in [139,140,199,204,205,206,207]).

Surprisingly, relatively little attention has been paid to the role of AA in CNS development and maturation [138]. AA is known to influence the myelination and differentiation of neuronal progenitors [208,209,210,211,212,213]. Equally important is the modulation exerted by AA at the DNA level during brain development [206,214,215]. It is of interest to note that the targeted deletion of SVCT2 (the major AA-specific transporter in the brain) and the consequent diminishment of brain AA to low-to-undetected levels result in death of newborn mice shortly after birth [138], thus indicating a major role of AA in the development of the brain and CNS function. 

On the other hand of the developmental spectrum, plasma AA might be associated with improved memory and cognition in aged populations and, in fact, may reduce the incidence of AD, although this remains controversial [216]. Interestingly, the ex vivo uptake of AA is retarded in acute hippocampal slices in aged rats; consequently, even exogenous application of AA does not alleviate oxidative stress in these animals [217]. Further evidence for a relationship between brain AA uptake and metabolism in exacerbating ageing-related pathologies of the brain has come from two recent studies. The first study provided evidence for impaired SCVT2 functions and a consequent deficiency in the neuronal uptake of AA in cellular and mouse models of Huntington’s disease [196]. The second study illustrated increased susceptibility of a transgenic mouse model of AD to kainic acid-induced seizures by decrement in AA uptake mediated by the partial knockout of SCVT2 [218]. Data from these studies suggest that a disruption in AA uptake and metabolism in the brain may be a common feature of ageing-related neurodegeneration. It is of note that AA was found to concomitantly instigate hippocampal neurogenesis in an experimental rat model of d-galactose-induced brain ageing with the restoration of hippocampal-dependent memory functions [219]. Indeed, aged human subjects with dementia and AD-like symptoms have reduced plasma AA levels [220,221], and supplementation with AA may be beneficial in reducing the risk for the development of AD [222]. Interestingly, in a study conducted in hospitalized, acutely-ill aged patients, AA deficiency was found to be significantly associated with symptoms of depression [223], indicating that AA deficiency in ageing may elicit multiple neurocognitive consequences.

## 7. AA as a Potential Ameliorative Agent in Pb Neurotoxicity

**Figure 3:**
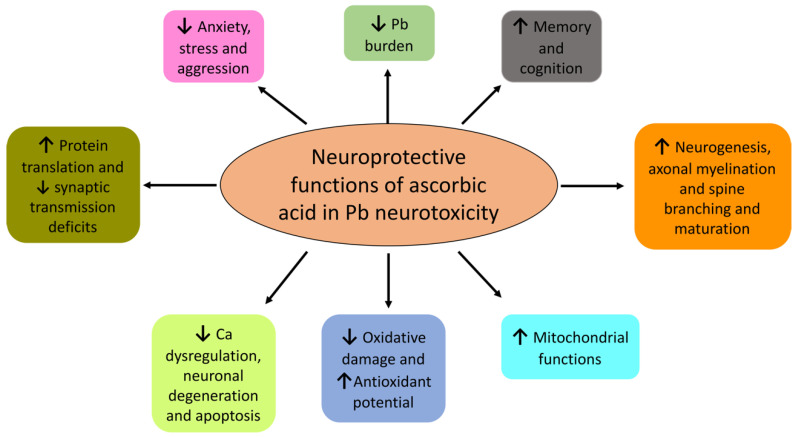
Mechanisms of ascorbic acid-mediated neuroprotection in Pb neurotoxicity. Stemming from its ability for metal ion chelation, antioxidant potential, and neuromodulation function, ascorbic acid attenuates Pb neurotoxicity in a multimodal manner, ranging from decreasing the Pb burden to alleviating biochemical and neurobehavioral deficits.

How effective is AA against Pb neurotoxicity? Mechanistically, AA can influence Pb-induced effects by multiple means, e.g., the chelation of Pb^2+^ ions that results in both reduced absorption in the gut and increased renal excretion. Is it of note that AA fulfils all the criteria for being an effective chelator against Pb^2+^ due to its (1) water solubility, (2) resistance to metabolic degradation, (3) ability to access metal storage sites, (4) prompt renal excretion, (5) retention of its chelation ability at a physiological pH, and (6) ability to reduce the toxicity of the metal upon formation of a complex with it [224]. Animal studies have supported the idea that orally administrated AA can chelate Pb and both increase its urinary clearance and reduce its intestinal absorption [225,226,227,228], as detailed in Section 7.2. Moreover, AA also reverses oxidative damage induced by Pb (see Section 4). In fact, AA also fulfils all the three criteria for acting as an effective antioxidant in Pb poisoning due to its abilities to (1) retard generation of free radicals and inactivate radical chain reaction; (2) chelate Pb^2+^ and prevent further radical generation; and (3) activate other endogenous antioxidant systems [229]. Indeed, animal studies have provided unequivocal evidence that AA both reduces oxidative damage and augments endogenous antioxidant potential in events of Pb exposure. Because of its multimodal effects, AA has unsurprisingly been proposed as a potential detoxifying agent in Pb toxicity (Figure 3) [4,229,230,231].

What are the advantages of AA over commonly used therapeutic agents against Pb neurotoxicity? The most commonly used antidotes for Pb toxicity are metal chelators, calcium disodium ethylene diamine tetra-acetic acid (CaNa_2_EDTA), and 2,3-meso-dimercaptosuccinic acid (succimer) [232,233]. However, the usage of these agents is rife with problems, such as delivery, bioavailability, and non-specific actions and toxicity. Hence, they may not be suitable in high-dose and long-term treatments [226,232,233,234,235,236,237]. This issue is especially important for cases where the subjects cannot be moved away from the sources of poisoning. Moreover, the use of chelators is not recommended in children, particularly for mild Pb exposure, which comprises majority of the cases [58,234]. In fact, chelation therapy in Pb-exposed children has not shown any benefits in reducing BLLs over time or stimulating cognitive, behavioral, and neuromotor attributes [24,238,239]. Interestingly, the use of succimer during pregnancy might actually enhance Pb-induced fetal developmental toxicity in mice, while Ca and AA reduce the maternal transfer of Pb to a fetus [240]. Hence, more attention should be given to safe and efficient natural dietary supplements. AA excellently fulfils the criteria in these respects.

### 7.1. Human Studies

Many human studies have provided evidence for the suitability of using AA as an ameliorative therapeutic agent in Pb exposure. Interestingly, the beneficial effects of AA in human Pb poisoning cases have been known since late 1930s. Thus, two case studies reported significant clinical improvements among a population of workers with occupational Pb exposure after the daily administration of AA [241,242]. Much later, Papaioannou et al. reported a reduction in BLLs in battery industry workers upon supplementation with 2 g of AA and 60 mg of zinc per day—importantly, while the workers were still on the job and constantly exposed to a Pb-rich environment [243]. Consistent with those studies, the amelioration of occupational Pb toxicity in a small sample of 36 Indian battery workers by AA supplementation (500 mg/day for a month) was observed, as evidenced by the reduction in the oxidative stress markers of lipid peroxides and nitrites in serum and the stimulation of antioxidant parameters, erythrocyte superoxide dismutase, and catalase; however and surprisingly, these effects occurred with no reduction in BLLs [244]. Ademuyiwa’s group conducted a series of studies on the effects of AA supplementation in Nigerian workers who were occupationally exposed to Pb. AA administration to auto-mechanics and attendants in petrol stations at a daily dose of 500 mg for two weeks resulted in marked decreases in BLLs, incidentally with increased urinary Pb excretion [245,246,247,248]. The AA-mediated reversal of the biochemical effects of Pb toxicity—such as reversal of elevated plasma, urine δ-aminolaevulinic acid (ALA), and glutathione levels; inhibition of ALAD; increased erythrocyte protoporphyrin; amelioration of ionoreglatory disruptions; inhibition of erythrocyte Ca^2+^-Mg^2+^-ATPase; and alterations in plasma Ca^2+^ and Mg^2+^ levels—have also been observed [245,246,247]. In a more recent study conducted in Mexican workers occupationally exposed to Pb, prolonged administration of AA significantly lowered BLL; restored blood ALAD activity, total antioxidant capacity, and the activities of antioxidant enzymes (such as superoxide dismutase (SOD), glutathione reductase (GR), and glutathione peroxidase (GPx)); and repressed blood oxidative damage, as assessed by lipid peroxidation products [249]. Pb-induced testicular dysfunction, as assessed by sperm parameters and sperm DNA fragmentation in workers from the battery-manufacturing industry from India, was found to be significantly reduced by AA supplementation at a dose of 1000 mg/day, five days per week, for a duration of three months [250]. Oral administration of 500 mg of AA daily for a month resulted in marked decreases in BLLs in Indian silver refiners who were occupationally exposed to Pb, in parallel with the reversal of the inhibition of the blood ALAD activity and increased blood Cu and Fe levels [251].

AA’s relationship with Pb in human subjects with nonoccupational and mild Pb exposure (including pregnant women and children) has also been characterized. For example, Sohler et al. conducted an uncontrolled trial and observed the effects of combined zinc and AA administration with a marked reduction of BLLs in a population of over 1000 psychiatric outpatients [252]. AA was also found to decrease BLLs in 85 subjects who volunteered to consume Pb-spiked drinks [253]. In another study conducted on heavy smokers, AA administration for a week had a pronounced (>80%) decrease in BLLs, but urinary Pb levels were not affected, compelling the authors to propose a reduced absorption of Pb [254]. Two other studies observed mild but nonsignificant decreases in BLLs upon the administration of AA [255,256], possibly due to the small sample size of their respective studies. After adjusting for age, education level, smoking, and alcohol consumption, the dietary intake of AA was found to be inversely correlated with BLLs in a population of over 700 male adults aged between 49 and 93 [257]. In one of the most prominent studies, Simon and Hudes conducted a large scale (over 19,000 subjects) population-based study in both American youths and adults, and they reported an independent inverse relationship between serum AA and BLLs among subjects [258], thus suggesting that a higher intake of AA may be effective in preventing Pb toxicity. Importantly, adults in the upper two quartiles of serum AA levels were found to have 65% and 68% less chances of eliciting high BLLs. Moreover, children in the highest serum AA quartile were found to be 89% less likely to have increased BLLs when compared to children in the lowest quartile for AA levels [258]. This study made the first significant contribution to the analysis of a relationship between AA and Pb with a large human sample size [259]. In another study with pregnant women, a significant reduction in BLLs was observed upon vitamin and mineral supplementation, with negative correlations between blood AA levels and BLLs in both supplemented and non-supplemented women [260]. It has been suggested that AA as a nutritional factor may be critical in the determination of BLLs in young children [259]. Indeed, AA supplementation has been shown to reduce the Pb burden in rural children, as assessed by Pb content in their hair samples, possibly by increasing the urinary excretion of the Pb–ascorbate complex [231]. In pregnant women, the supplementation of AA in combination with calcium phosphate was found to reduce the Pb burden in the placenta by 90% and in mothers’ milk by 15% [261]. In an urban population of Korean adults with the supplementation of dietary antioxidants, an increase in AA levels was significantly correlated with decrease in BLLs and the urinary oxidative stress marker 8-hydroxy-2′-deoxyguanosine (8-OHdG) [262]. In an interesting study combining clinical and animal data, Jin et al. observed significant ameliorative effects of AA (in combination with succimer and calcium supplementation) in early-life, mild Pb toxicity with significant reversals of Pb burdens in blood and bones and blood ALAD activity [263].

### 7.2. Animal Models: Reducing Pb Burden and Amelioration of Neuropathological Defects

Among the first studies that probed the effectiveness of AA as a prophylactic agent in reducing the Pb burden in experimental animals, Rudra et al. found partial rescue effects of AA supplementation in Pb-poisoned rats in terms of Pb burden and AA metabolism [264]. Suzuki and Yoshida found that dietary AA (upon co-administration with Fe) reduced the Pb burden in the liver, kidneys, and tibia bone, and it also reduced anemia and growth deficits in growing rats challenged with Pb for short [265] and moderately long terms [266], although the curative effects in the long term regime were only modest. An experimental model of chicks surprisingly did not show any interaction between AA and Pb [267]. Goyer and Cherian observed an increased urinary excretion of Pb and a reduced Pb burden in the blood, bone, brain, kidney, and liver in Pb-exposed, one-month-old rats treated with a combination of AA and EDTA [268]. An independent study also confirmed AA-mediated stimulation of the renal clearance of Pb and a consequent decrease in BLLs in rats [225], attesting to the proposed role of AA as a potential therapeutic agent in Pb poisoning. Consistently, a number of publications by Tondon and co-authors in the late 1980s also provided evidence supporting the effects of AA as a protective agent as part of a combinatorial supplementary regime on the reduction of the whole body Pb burden in multiple organs of experimental animals challenged with Pb [224,269,270]. In a study conducted in New Zealand, Dalley et al. found a strong prophylactic effect of AA supplementation in reducing the Pb burden in the femur bone, liver, kidney, and plasma of rats challenged with intravenous Pb [226]. A combinatorial therapeutic strategy against Pb exposure in drinking water in mice consisting of a supplementation of succimer, Ca, and AA was found to achieve significant ameliorative effects on the mobilization of blood, renal, hepatic, and brain Pb, as well as blood ALAD activity [271]. Collectively, data from these studies indicate that AA, particularly as part of a combinatorial therapeutic strategy, is efficient in reducing the Pb burden in multiple organs in animals challenged with Pb insults.

Recent studies have extended the knowledge base of AA–Pb interactions with respect to the ameliorative mechanisms involved at the behavioral, electrophysiological, and biochemical levels. For example, chronic intraperitoneal infusion of AA was found to rescue electrophysiological deficits in synaptic transmission (both spontaneous and evoked) in the neuromuscular junctions in the dorsiflexor skeletal muscle of mice exposed to Pb, thus indicating the protective roles of AA against Pb in peripheral nerves [272]. AA has also been shown to attenuate Pb-induced deficits in neurobehavior, such as memory-dependent cognitive functions including novel object recognition [273], anxiety, and aggression [274]. The assessment of a range of antioxidant nutrients for their effectiveness in reducing the Pb burden in adult mice identified AA as one of the promising chelating antioxidant nutrients with the capacity to reduce BBLs to almost 60% without any adverse effects on Ca, Fe, and Zn absorption [275]. The amelioration of Pb-induced oxidative stress in rat brains has also been proposed in other studies [230,274,276,277,278].

### 7.3. Animal Models: Developing Brain

Several animal studies have indicated that AA may serve as an effective ameliorative agent in events of gestational and lactational exposure to Pb. AA, as part of a dietary supplemental regime, was recently shown to reduce BLLs and reverse serum ALAD activity levels in juvenile mice pre-exposed to Pb with the consequent alleviation of deficits in behavior and redox homeostasis, importantly without eliciting any unwanted side-effects [233]. In juvenile rats co-exposed to lead and chlorpyrifos, an organophosphate insecticide, the administration of AA resulted in a marked reduction in oxidative damage concomitantly with the reversal of neurobehavioral and sensorimotor deficits [279]. An enriched milk formula consisting of AA and other antioxidants reduced BLLs and the Pb burden in organs such as the liver, kidney, bones, and brain in mouse pups exposed to Pb, along with ameliorative effects on blood ALAD, protoporphyrin, and thiobarbituric acid reactive substances (TBARS) levels [280]. The AA-mediated rescue of brain oxidative damage induced by perinatal exposure to Pb in rat pups was confirmed upon coadministration with a mixture of antioxidants including vitamin C and E during pregnancy and lactation [229]. In the follow-up study, the authors found further evidence for AA as a potential therapeutic agent with the partial reversal of deficits in ALAD activity, as well as the activity of catalase enzyme and levels of TBARS, in the same model of early-life Pb exposure [281]. In addition to lowering the Pb burden in the brain, Ghasemi et al. found neuroprotective effects of AA supplementation in juvenile rats exposed to Pb on oxidative stress markers, oxidized thiols, and TBARS, as well as spatial memory tested in a Morris water maze and passive avoidance learning [282,283]. Memory deficits in rat pups induced by chronic Pb exposure during gestation and lactation have been shown to be attenuated by AA and AA-rich traditional medicine lemon balm (*Melissa officianlis*) with comparable prophylactic effects, indicating AA as the major neuroprotective agent in *Melissa* [284]. A histopathological evaluation of young adult rats revealed that the intragastric intubation and oral supplementation of AA rescued Pb-induced effects on brain edema, satellitosis, monocytic aggregation, and encephalomalacia [285]. In an early-life Pb exposure model with gestational and lactational Pb exposure, AA in combination with fresh garlic juice extract was found to efficiently reduce blood and brain Pb levels, as well as to attenuate the detrimental effects of Pb-induced neurogenesis, as observed by doublecortin immunostaining [286]. Concomitant with a reduction in BLLs, retinal apoptosis, as assessed by terminal deoxynucleotidyl transferase dUTP nick end labeling (TUNEL) staining was found to be significantly attenuated by AA alone and with the coadministration with garlic juice extract in a developmental model of Pb toxicity in rat pups [287]. Moreover, there were AA-mediated protective effects on hippocampal long-term potentiation (LTP), with both population spike amplitude and slope of excitatory postsynaptic potentials), possibly due to reduced oxidative stress and an increase in antioxidant potential in rats challenged with Pb [288]. Consistently, AA alone or in combination with vitamin E was found to reduce oxidative damage and rescue deficits in endogenous antioxidant enzymes, such as GPx and SOD, in the hippocampi of rats challenged with Pb. In addition, the authors observed the attenuation of Pb-induced dysfunction in nitric oxide synthase (NOS) [289]. The preventive effects of AA in young adult rats challenged with Pb on biochemical antioxidant and signaling components (SOD, NOS, NO, Ca2+/calmodulin-dependent protein kinase II (CamKII), and cAMP response element binding protein (CREB)) in the hippocampus were reported and paralleled with the amelioration of spatial memory deficits in a Morris water maze test [290]. Other studies have also found ameliorative effects of AA alone and upon coadministration with a garlic juice extract on hippocampal apoptosis and neuronal degeneration in all the three major subregions (CA1, CA3, and dentate gyrus) examined and in a gestational and lactational model of Pb toxicity in rats [291,292].

Our own studies have provided evidence for the amelioration of early-life (perinatal and postnatal—from gestational day (GD) 15 to postnatal day (PND) 21) Pb-mediated effects on the nerve terminals of multiple brain regions, such as cerebral [293], hippocampal [293], and cerebellar synapses [294], by maternal AA supplementation. In particular, we observed a marked reversal of Pb-mediated alterations in synaptic bioenergetics with the rescue of defects in the mitochondrial membrane potential (MMP) and activities of the enzymes of the electron transport chain (ETC) [294,295]. Improvements in endogenous antioxidant pathways (particularly glutathione signaling) concomitantly with reduced oxidative damage to proteins and lipids were also observed in our model of AA supplementation in rats developmentally exposed to Pb [294,295]. An independent, recently published study also observed synergistic actions of AA and *Schisandra chinensis* extracts, but not AA alone, in the positive modulation of mitochondrial respiration in normal, wild-type mice [296]. Of note, we also provided evidence for altered de novo hippocampal protein translation in rats with early-life Pb exposure, mediated by deficits in signaling through the protein kinase Akt pathway [293]. These effects were also shown to be rescued by treatment with AA [293], which is consistent with other studies that have proposed positive modulatory actions of AA on hippocampal Akt signaling. For example, Fraga et al. found that AA supplementation in unchallenged rats resulted in the upregulation of hippocampal synaptic proteins such as synapsin, as well as increased dendritic spine density, maturation, and performance, in a novelty suppressed feeding test through the activation of the Akt pathway and its downstream effector 70S6K [297], which phosphorylates and regulates the function of ribosomal protein S6, a component of the translational machinery. Moreover, anti-depressant-like effects elicited by the oral delivery of AA have been shown to be mediated by the activation of phosphoinositide 3-kinase (PI3K) and Akt signaling, as well as the consequent activation of 70S6K that occurs simultaneously with a sharp elevation in postsynaptic density protein 95 (PSD-95) levels in the hippocampi of two-month-old young adult mice [298]. AA has also been shown to partially reverse the detrimental effects of maternal Pb exposure in rat pups on the morphology of hippocampal CA1 neurons, as assessed by Golgi staining and Sholl’s analysis [299].

In a series of studies, an independent contemporary research group based in South Korea also provided further evidence in favor of using AA as a therapeutic agent in developmental Pb neurotoxicity, particularly its effects on cellular apoptosis, endogenous antioxidant enzymes, synaptic dysfunction, and axonal myelination. Their data suggest that AA induces marked stimulation of the endogenous antioxidant enzymes Mn and Cu/Zn SODs and catalase in the hippocampi of rat pups that were gestationally and postnatally exposed to Pb, in parallel with the rescue of degenerating neurons and attenuation of the induction of the pro-apoptotic protein Bax [300,301]. In follow-up studies focusing on cerebellar cortices, the authors observed appreciable rescue of cellular degeneration, as assessed by TUNEL staining by AA administration, concomitantly with the amelioration of the Pb-induced induction of apoptotic protein Bax [302] and degenerative changes such as the reduction of glutamic acid decarboxylase 67 (GAD67) and receptor tyrosine kinase c-kit proteins [303]. The authors further provided evidence for AA-mediated attenuation of Pb-induced effects on glutamatergic signaling and oxidative stress, as assessed by the expression of the synaptic proteins synaptophysin, PSD95, NMDA receptor subunit 1 (NMDAR1), and brain-derived neurotrophic factor (BDNF), as well as antioxidant SODs, in a developmental Pb exposure model [304,305]. Furthermore, reversal of Pb-induced effects on Purkinje cells in parallel with rescue of Pb-induced alterations in calcium binding proteins such as calbindin, calretinin, and parvalbumin, as well as γ-aminobutyric acid transporter 1 (GABAT1) [306], osteopontin, Olig2-immunorecative oligodendrocytes, and axonal myelination (assessed by the expression of myelin-associated glycoprotein (MAG) and myelin basic protein (MBP)) upon AA supplementation was also reported [305,307]. A recent study corroborates the attenuation of toxicity of early-life (gestational and lactational) Pb exposure in the cerebellum of rat pups upon the supplementation of AA by oral gavage by using the assessments of oxidative stress markers and the antioxidant system, as well as histopathological examinations and tests for neuromotor functions (forelimb grip and negative geotaxis) [308]. Interestingly, ameliorative effects of AA in Pb poisoning in the cerebellum of adult rats have also been proposed [309].

## 8. Other Non-Neuronal Attenuative Effects of AA in Pb Poisoning

It should be noted that the effect of AA in the restoration of Pb-induced toxicity is not limited to the brain. Indeed, the non-neuronal amelioration of Pb-mediated effects in experimental animals by AA either alone or in combination with other proposed neuroprotective agents has been observed in blood biochemistry and hematological parameters [265,306,307,308,309,310,311,312,313,314,315,316], cardiac functions [317], hepatic physiology [277,285,312,318,319,320,321,322,323,324], renal functions [285,310,312,325,326], the colon [327], testicular functions and spermatogenesis [285,328,329,330,331,332], sperm morphology and physiology [333], thyroid hormone synthesis [334], the lungs [335], and clastogenicity in bone marrow cells [336,337,338]. Readers are suggested to refer to a recent review paper [339] for a brief overview of the non-neuronal effects of AA in Pb poisoning.

In vitro cell culture systems have also been employed to successfully access the ameliorative effects of AA on Pb uptake and release [340], Pb-induced genotoxicity and death [341,342], xenobiotics metabolism [343], and oxidative damage to cellular lipids, the nuclear factor (erythroid-derived 2) like 2–Kelch-like ECH-associated protein 1 (Nrf2–KEAP1) antioxidant pathway, and that pathway’s downstream enzyme effectors [344].

Finally, the dietary supplementation of AA has also been shown to be effective in Pb poisoning in other experimentational animals such as sea cucumbers (*Apostichopus japonicus*) [345], rockfish (*Sebastes schlehelii*) [346], egg-laying hens [347], and rabbits (*Oryctolagus cuniculus*) [348,349].

## 9. Conclusions

As a safe, readily available, and low-cost supplemental biomolecule, ascorbic acid might prove to be a useful ameliorative therapeutic agent against Pb toxicity, both in reducing BLLs and reversing the effects of Pb poisoning. This is particularly true in children, where prominent chelation therapeutic agents have largely failed. It should be noted that most of the recent studies delineating the effects of AA on Pb neurotoxicity have come from developing countries. This is expected because Pb toxicity is a major concern in these countries in particular because of their emerging industrial potential and issues such as a lack of proper legislation and compliance that aggravate the conditions. This can be related to recent outbreak of a ‘mystery’ illness that grasped several hundreds of individuals, including children, with symptoms such as nausea and seizures in the Eluru district of southern Indian state of Andhra Pradesh. A preliminary investigation of blood samples of the subjects indicated high level of contamination with heavy metals, particularly Pb and Ni. Our understanding of AA–Pb interactions is nevertheless still limited, and we are far from fully appreciating the usefulness of this vitamin as an effective ameliorative agent. Further studies are hence required (1) to confirm the prophylactic effects of AA in both animal models and human subjects with Pb exposure, as well as (2) to understand the mechanisms of protection offered by AA. With this review, we hope to rekindle research interest in this essential, water-soluble, and practically harmless vitamin as an effective neuroprotective agent, not only in developmental Pb neurotoxicity but also in other neuropathologies.

## Abbreviations

70S6K	S6 kinase 70 kDa
8-OHdG	8-hydroxy-2′-deoxyguanosine
AA	ascorbic acid; AD: Alzheimer’s disease
ADHD	attention deficit hyperactivity disorder
ALA	δ-aminolaevulinic acid
ALAD	δ-aminolaevulinic acid dehydratase
ASD	autism spectrum disorder
BBB	blood–brain barrier
BLL	blood lead level
CamKII	Ca^2+^/calmodulin-dependent protein kinase II
CaNa_2_EDTA	calcium disodium ethylenediaminetetraacetic acid
CREB	cAMP response element binding protein
CSF	cerebrospinal fluid
ECF	extracellular fluid
EECHO	environmental exposures and child health outcomes
ETC	electron transport chain
GABAT1	γ-aminobutyric acid transporter 1
GAD67	glutamic acid decarboxylase 67 kDa
GD	gestational day
GLUT1	glucose transporter 1
GPx	glutathione peroxidase
GR	glutathione reductase
HPA	hypothalamic pituitary adrenal
IQ	intelligence quotient
KEAP1	Kelch-like ECH-associated protein
LTP	long-term potentiation
MAG	myelin-associated glycoprotein
MBP	myelin basic protein
mDISC1	mutated-in-schizophrenia 1
MMP	mitochondrial membrane potential
NMDAR1	*N*-methyl-D-aspartate receptor 1
NOS	nitric oxide synthase
Nrf2	nuclear factor (erythroid-derived 2)-like 2
Pb	Lead (plumbum)
PI3K	phosphoinositide 3-kinase
PND	postnatal day
PSD95	postsynaptic density protein 95
SOD	superoxide dismutase
SVCT1	sodium-dependent vitamin C transporter 1
SVCT2	sodium-dependent vitamin C transporter 1
TBARS	thiobarbituric acid reactive substances
VDR	vitamin D receptor
